# Effects of cooking methods on starch and sugar composition of sweetpotato storage roots

**DOI:** 10.1371/journal.pone.0182604

**Published:** 2017-08-21

**Authors:** Shuying Wei, Guoquan Lu, Heping Cao

**Affiliations:** 1 College of Agriculture & Food Science and The Key Laboratory for Quality Improvement of Agricultural Products of Zhejiang Province, Zhejiang A & F University, Lin'an, Zhejiang, China; 2 U.S. Department of Agriculture, Agricultural Research Service, Southern Regional Research Center, Commodity Utilization Research Unit, New Orleans, Louisiana, United States of America; Agriculture and Agri-Food Canada, CANADA

## Abstract

Sweetpotato has rich nutrition, good ecological adaptability and high yield. There is a lack of knowledge about the effects of cooking methods on starch and sugar components in elite Chinese cultivars. In this study, sweetpotato storage roots from four cultivars “*Xinxiang*”, “*Jinyu*”, “*Zimei*” and “*Yuzishu 263*” were treated by baking, boiling and steaming and subsequently analyzed for starch content, amylase activity and sugar contents including glucose, fructose, sucrose and maltose. Results indicated that cooking reduced starch content and final amylase activity and increased reducing sugar content especially maltose content, but did not have significant influence on non-reducing sugar content. These effects were different among the four cultivars and three cooking methods. Baking led to the least starch reduction. Storage roots of “*Jinyu*” contained the highest amount of sugar content and thus sweetest. Sugar composition analysis suggested that cultivars “*Xinxiang*” and “*Jinyu*” belong to high-maltose cultivars. This study may provide useful information for evaluating the cooking quality of sweetpotato cultivars.

## Introduction

Sweetpotato (*Ipomoea batatas*), a dicotyledonous plant, belongs to the *Convolvulaceae* family. It is a root vegetable due to its large, starchy, sweet-tasting storage roots. Sweetpotato is native to the tropical regions in America. It is distantly related to common potato (*Solanum tuberosum*).

Sweetpotato has rich nutrition [1], good ecological adaptability [2] and high yield [3]. The edible storage roots contain high sugar content, desirable cooking odor, and multiple bioactive compounds with nutritional and health benefits [4–6]. Existing research has shown that soluble sugar composition in raw sweetpotatoes includes sucrose, glucose and fructose, while maltose is specifically found in cooked sweetpotatoes [7–13].

One of the most important edible quality parameters of sweetpotatoes is sweetness [14,15]. Sweetness of raw sweetpotatoes has been considered as an index for cultivar evaluation [16,17]. However, some studies have shown that sweetness has little correlation with soluble sugar content of raw sweetpotatoes, but largely related to sugar content of cooked sweetpotatoes [18,19]. The sugar compositions in raw and cooked sweetpotatoes were reported using varieties from the Ghana [11], US [10], Philippine [12], and Tanzania [8]. However, it was less known about the effects of cooking methods on starch and sugar components among the elite cultivars in China.

The objective of this study was to provide information on the effects of cooking methods on carbohydrate composition and its relation to cultivar appraisal of sweetpotatoes. Here we reported the determination of sugar composition with HPLC and analysis of the difference of starch and sugar components among four sweetpotato cultivars under three cooking treatments.

## Materials and methods

### Materials

Sweetpotato cultivars selected in the study were representatives of the yellow flesh cultivars (“*Xinxiang”* and “*Jinyu”*) and the purple flesh cultivars (“*Zimei”* and “*Yuzishu263”*). “*XinXiang*” and “*Jinyu*” were created by Agricultural Academic Science Institution of Zhejiang Province. “*XinXiang*” storage root is red skin and oval shape. “*Jinyu*” storage root is red skin and long. “*Yuzishu 263*” was bred by Sweetpotato Institution of Southwest University in Chongqing City, and the storage root is purple skin and looks long. “*Zimei*” was introduced from Vietnam, and the storage root is purple skin and oval shape.

All storage roots were freshly harvested within one week before being used in the experiments. The same sizes of storage roots (about 250 g) were used in the study. Chemicals (such as methanol, acetonitrile, HPLC grade) were purchased from East China Medicine Company.

### Methods

#### Cooking methods

Boiling: sweetpotato storage roots were washed with water, soaked into water in a pan followed by heating them using electric cooker. It took 28 min for boiling in boiled water. Steaming: sweetpotato storage roots were put on steaming box above the water line in the pan. It also took 28 min for steaming since water boiled. Baking: sweetpotato storage roots were settled in a baking box, set temperature at 200°C. It took 45 min for baking at 200°C. We did not peel the storage roots and performed any cutting before cooking. After cooking, samples were cooled to normal temperature, and cut into pieces (1.0 cm*1.0 cm*0.2 cm). Some of them were freezing drought under -45°C for 48 hr before being smashed; others were baked at 80°C until the weight kept consistent. Raw sweetpotatoes (as control treatment) were also cut into pieces (1.0 cm*1.0 cm*0.2 cm) before drought as above.

#### Bake-dried rate and freeze-dried rate determination

Bake-dried rate and freeze-dried rate were calculated as follows: Bake-dried rate = baked sugar content (g) / bake-dried weight (g)*100%. Freeze-dried rate = freeze-dried sugar content (g) / freeze-dried weight (g)*100%.

#### Sugar composition analysis

Soluble sugars were extracted from the sweetpotato samples based on the published method [10,14] with minor modifications: 5 g sample of each sweetpotato mash was added to a sealed test tube. 5 mL of 80% ethanol was added to each sample and mixed. The test tube was placed in a water bath at 80°C for 15 min. Subsequently, 2.5 mL of 80% ethanol was added at 15 and 30 min. Fresh 80% ethanol was then added to bring the total volume to 10 mL. The mixture was centrifuged under 8000 *rpm* for 15 min. The supernatant was collected, filtered through a 0.45-μm membrane filter and injected into a high performance liquid chromatography (HPLC, Agilent 1200) with a refractive index detector. HPLC employed reverse-phase C-18 column (XBridgeTM Amide 3.5 μm, 4.6×250 mm); sample volume was 10 μL; mobile phase was 70% acetonitrile:H_2_O (7:3); and flowing rate was 0.5 mL/min. The column was heated to 35°C.

#### Sweetness analysis

Sweetpotato sweetness was calculated according to the method [20] using the following formula (where x is soluble sugar and y is sweetness): Raw sweetpotato: y = 0.0048x +0.0328, r = 0.962. Cooked sweetpotato: y = 0.0170x +0.0766, r = 0.977.

#### Starch content determination

Starch was prepared and detected based on the method of Cao [21]. It was degraded into sugar by 9 mol/L perchloric acid (AR, supplied by East China Medicine Company) in 100°C for 30 min. Starch degradation rate = [untreated starch content (g)—treated starch content (g)] / untreated starch content (g)*100%.

#### Amylase activity determination

α-amylase activities were determined by the starch azure assay with minor modification [22]. The sample (0.5 g) was homogenized in a Mortar in 5 mL extraction buffer containing 100 mM potassium phosphate buffer (pH 7.0), 5mM ethylenediaminetetraacetic acid (EDTA) and 1mM dithiothreitol (DDT). Following filtration and centrifugation at 8000*rpm* for 15 min, the supernatant was used to determine amylase activities.

#### Statistical analysis

All the analyses were done in triplicates. The data were subjected to statistical analysis using the statistical software SPSS13.1 and Microsoft Excel. Variance analysis had been applied, and Duncan’ test was also used.

## Results

### Effect of cooking methods on dry weight of sweetpotatoes

There was no statistical difference in dry weight between freeze-dried and bake-dried sweetpotatoes under the same cooking method, regardless of some variation (Table 1). Take “*Zimei*” as an example, under raw condition, the ratio of Dw/Fw (dry weight to fresh weight) between freeze-dried and bake-dried “*Zimei*” were 36.5% and 34.9%, while it was 38.2% and 38.5% under baked condition, 27.3% and 27.6% in boiling, and 33.3% and 35.3% in steaming. Every pair of data appeared approximately the same without statistical difference between the freeze-dried and bake-dried sweetpotatoes. Similarly, no significant difference on Dw/Fw was observed between freeze-dried and bake-dried sweetpotatoes for other cultivars under the same cooking method (Table 1). Boiling resulted in the lowest dry weight of storage roots among the three cooked and the untreated sweetpotatoes from all cultivars except “*Xinxiang*” (Table 1).

**Table 1:** Effect of cooking methods on dry weight of sweetpotato cultivars.

Variety	Raw sweetpotato		Baking		Boiling		Steaming	
	freezing dry	baking dry	freezing dry	baking dry	freezing dry	baking dry	freezing dry	baking dry
Jinyu	17.9 ± 1.0 ^a^	15.8 ± 0.6 ^a^	27.5 ± 0.5 ^a^	27.6 ± 0.7 ^a^	10.4 ± 0.4 ^a^	11.1 ± 0.7 ^a^	30.6 ± 1.0 ^a^	28.4 ± 0.8 ^a^
Xinxiang	24.7 ± 0.8 ^a^	23.4 ± 0.3 ^a^	33.6 ± 1.0 ^a^	36.9 ± 1.2 ^a^	28.4 ± 0.6 ^a^	27.8 ± 0.4 ^a^	27.6 ± 0.5 ^a^	29.3 ± 0.8 ^a^
Yuzishu263	35.6 ± 2.7 ^a^	32.5 ± 1.4 ^a^	36.8 ± 1.4 ^a^	33.1 ± 1.9 ^a^	22.5 ± 2.1 ^a^	26.3 ± 1.8 ^a^	27.5 ± 0.6 ^a^	28.1 ± 0.7 ^a^
Zimei	36.5 ± 0.8 ^a^	34.9 ± 0.5 ^a^	38.2 ± 1.1 ^a^	38.5 ± 1.0 ^a^	27.3 ± 0.7 ^a^	27.6 ± 0.6 ^a^	33.0 ± 0.7 ^a^	35.3 ± 0.8 ^a^

Note: The numbers in the table represent the dry weight as a percentage of fresh weight of raw sweetpotatoes and those after freezing dry and baking dry under different cooking conditions. Different lower-case letters besides the numbers in the table represent differences at 0.05 levels between freezing dry and baking dry under the same cooking treatment.

### Effect of cooking methods on starch content of sweetpotatoes

Among raw materials, “*Zimei*” and “*Yuzishu263*” had the highest starch content (189.4 and 175.4 mg/g.Dw, respectively), whereas “*Jinyu*” had the lowest (124.5 mg/g.Dw) (Table 2). In general, starch content decreased after cooking. Baking degraded starch by up to 20% and performed similar trend to raw sweetpotatoes. Steaming also reduced starch content of all cultivars especially “*Jinyu*” by more than one third, and “*Xinxiang*” by steaming had the lowest amount of starch. As to boiling, “*Jinyu*” and “*Zimei*” reduced starch content by 40–60%; “*Xinxiang*” and “*Yuzishu263*” had the highest amount of starch (Table 2).

**Table 2:** Effect of cooking methods on starch content of sweetpotato storage roots.

Variety	Raw sweetpotatoes	Baking (%)	Boiling (%)	Steaming (%)
Jinyu	124.5 ± 8.0 ^a^	103.6 ± 2.0 ^a^ (83.2)	52.5 ± 9.7 ^a^ (42.2)	125.5 ± 2.8 ^b^ (100.8)
Xinxiang	158.2 ± 2.6 ^b^	140.5 ± 4.4 ^b^ (88.8)	145.4 ± 5.0 ^c^ (91.9)	103.8 ± 3.1 ^a^ (65.6)
Yuzishu263	175.4 ± 5.2 ^c^	149.9 ± 2.2 ^c^ (85.5)	143.9 ± 5.4 ^c^ (82.0)	130.9 ± 3.6 ^b^ (74.6)
Zimei	189.4 ± 6.7 ^d^	150.6 ± 0.9 ^c^ (79.5)	117.8 ± 3.1 ^b^ (62.2)	161.4 ± 1.7 ^c^ (85.2)

Note: The numbers in the table represent the starch content (mg/g.Dw) of raw sweetpotatoes and those under different cooking conditions followed by the percentage of starch content in the cooked sweetpotatoes relative to those in raw sweetpotatoes. Different lower-case letters besides the numbers in the table represent significant difference at 0.05 levels among all cultivars under the same cooking treatment.

### HPLC separation of soluble sugars from sweetpotatoes

**Fig 1:**
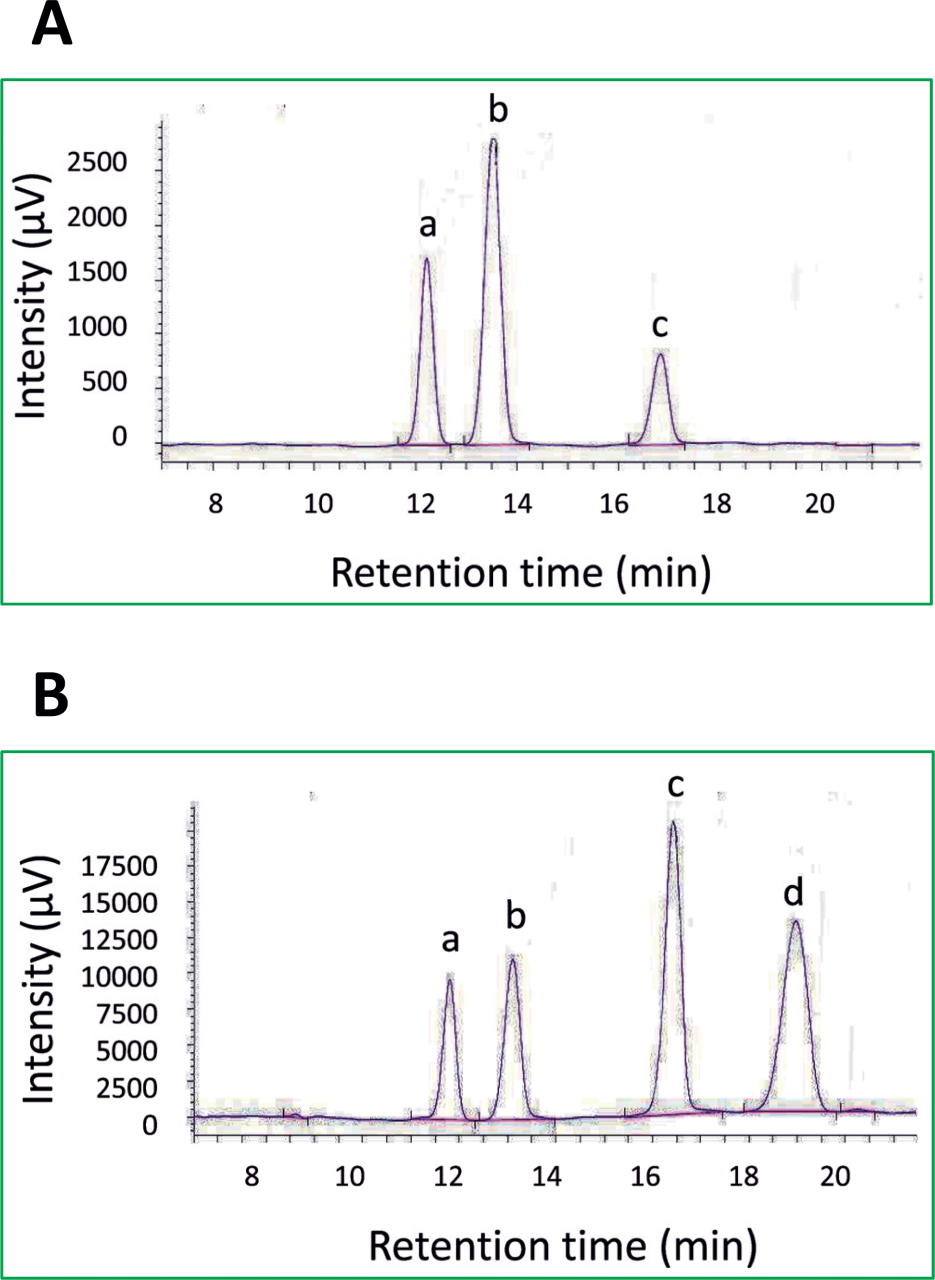
HPLC chromatograms of soluble sugars in sweetpotato storage roots. (A) raw sweetpotato storage roots from “*Xinxiang”*, (B) cooked sweetpotato storage roots from “*Xinxiang*.*”* a: fructose, b: glucose, c: sucrose, d: maltose.

HPLC was used to analyze the composition of soluble sugars in sweetpotato storage roots. Raw sweetpotato storage roots contained three soluble sugars: fructose (peak a), glucose (peak b) and sucrose (peak c) (Fig 1A). Cooked sweetpotato storage roots contained the same three sugars, as shown in the cultivar “*Xinxiang*” (peaks a-c) and generated extra maltose (peak d) (Fig 1B).

### Effect of cooking methods on sugar composition of sweetpotatoes

**Fig 2:**
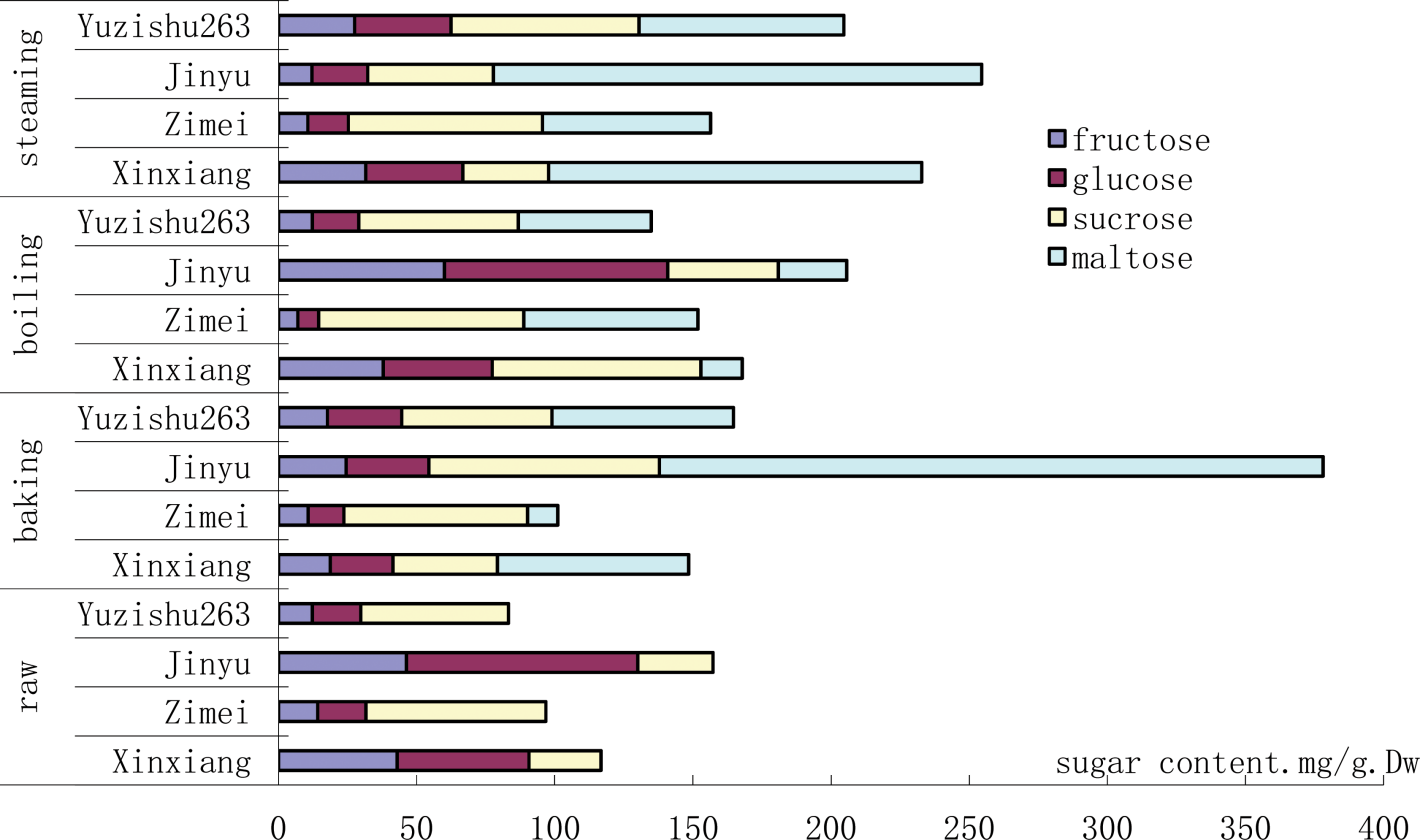
Effect of cooking methods on sugar composition in storage roots from different sweetpotato cultivars.

Cooking dramatically increased the content of soluble sugars, although it varied among cultivars (Fig 2). The highest content of soluble sugars in cooked sweetpotatoes was from “*Jinyu*”. The total sugar content in sweetpotatoes from “*Jinyu*” under baking, boiling and steaming were 378, 238 and 250 mg/g of dry weight, respectively. Most of the increased sugars were mainly due to maltose accumulation (Fig 2). Baking changed the order of sugar content in the sweetpotatoes from four cultivars as “*Jinyu*” > “*Yuzishu263*” > “*Xinxiang*” > “*Zimei*”. Maltose content in baked sweetpotatoes from “*Jinyu*” was increased the most by 240.2 mg/g of dry weight (Fig 2). Boiling also altered the profiles of sugar content and resulted in an order of “*Jinyu*” > “*Xinxiang*” > “*Yuzishu 263*” > “*Zimei*”. After boiling, maltose also increased significantly. Steaming resulted in a similar profile of sugars as boiling. After steaming, maltose was markedly detected in the treated samples. For example, maltose content in steamed sweetpotatoes from “*Xinxiang*” and “*Jinyu*” were increased respectively by 135 and 176.7 mg/g.Dw (Fig 2).

Raw sweetpotatoes from “*Jinyu*” contained the highest amount of sugars (159.4 mg/g.Dw) followed by “*Xinxiang*” (117.4 mg/g.Dw). Sucrose was the most abundant sugar in the raw sweetpotatoes of “*Yuzishu263”*and “*Zimei”* (Fig 2). Glucose was the most abundant sugar in raw “*Jinyu*” and “*Xinxiang*”, although fructose contributed significantly to their total amount of soluble sugars (Fig 2). In general, yellow flesh cultivars (“*Jinyu*” and “*Xinxiang*”) contained more sugars than purple flesh cultivars (“*Zimei”* and “*Yuzishu263*”). The order of total sugar content in raw sweetpotatoes were “*Jinyu*” > “*Xinxiang*” > “*Zimei*” > “*Yuzishu263*” (Fig 2).

### Effect of cooking methods on reducing and non-reducing sugars of sweetpotatoes

**Fig 3:**
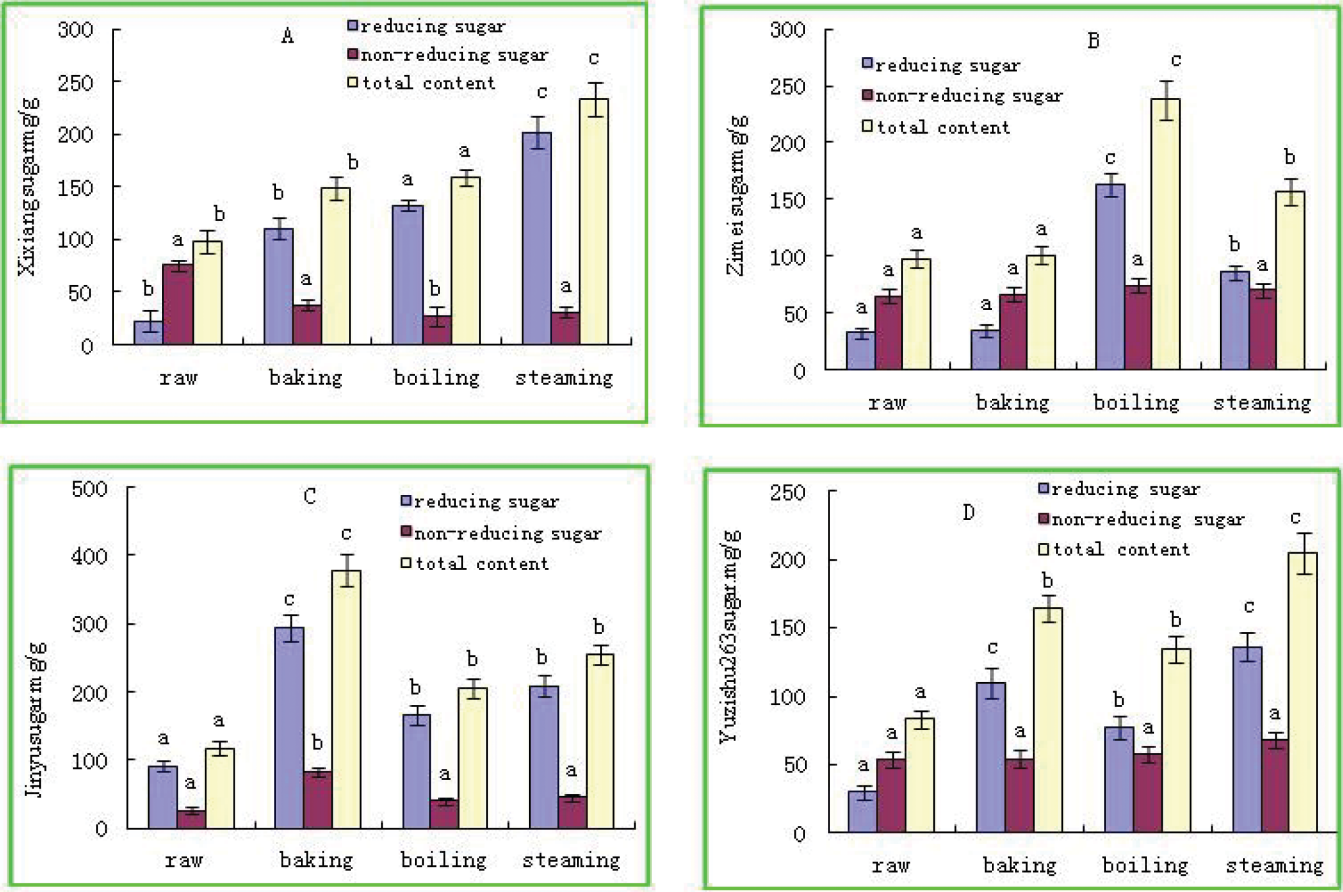
Effects of cooking methods on reducing and non-reducing sugars in storage roots from different sweetpotato cultivars. (A) cultivar “*Xinxiang*”, (B) cultivar “*Zimei*”, (C) cultivar “*Jinyu*”, (D) cultivar “*Yuzishu263*”. Different small letters represent significant difference at 0.05 levels of the same kind of sugar among all cultivars under the same treatment. “Total content” in the Figure means “total sugar content.”

Total soluble sugar content was increased by cooking, which was primarily due to the increases of reducing sugar (Fig 3). As to steaming, “*Xinxiang*” had the highest reducing sugar (210.7 mg/g.Dw) (Fig 3A). As to boiling, “*Zimei*” was the highest (163.6 mg/g.Dw) (Fig 3B). Under baking, the highest was “*Jinyu*” (294 mg/g.Dw) (Fig 3C). Among the four cooking methods, the steamed “*Yuzishu263*” cultivar had the highest reducing sugar content (136 mg/g.Dw) (Fig 3D). Non-reducing sugars of “*Jinyu*” increased one-fold by baking than by other cooking methods (Fig 3C). Except that, non-reducing sugar had little variation neither among cultivars nor cooking methods (Fig 3).

### Effect of cooking methods on sweetness

One important edible quality index of sweetpotatoes is sweetness, which is closely related to soluble sugars [23]. Therefore, we analyzed sugar concentration to calculate sweetness in order to facilitate quality analysis and cultivar appreciation (Table 3). Sweetness of cooked sweetpotatoes had increased significantly compared to raw sweetpotatoes, and there were also significant differences among the cultivars. Sweetness of “*Jinyu*” was the highest either in raw sweetpotatoes (index 0.77) or under baking (index 6.5) and steaming (index 4.4). By baking, boiling and steaming, sweetness of “*Jinyu*” increased respectively by 8.44-, 4.64- and 5.71-fold, compared to raw state (Table 3). Sweetness of boiled “*Zimei*” and steamed “*Xinxiang*” were also high (index more than 4) but boiled “*Xinxiang*” turned to be the least (index 1.75) (Table 3). By baking, boiling and steaming, sweetness of “*Xinxiang*” increased respectively by 4.19-, 2.82- and 6.50-fold (Table 3). Meanwhile, under the above three treatments, sweetness of “*Yuzishu263*” increased by 6.74-, 5.51- and 8.37-fold (Table 3), while “*Zimei*” increased by 3.60-, 8.24- and 5.48-fold, respectively (Table 3).

**Table 3:** Effect of cooking methods on sweetness of sweetpotato storage roots.

Variety	Raw sweetpotatoes (%)	Baking (fold)	Boiling (fold)	Steaming (fold)
Jinyu	0.77 ± 0.03 ^d^ (100)	6.50 ± 0.12 ^c^ (8.44)	3.57 ± 0.09 ^c^ (4.64)	4.40 ± 0.15 ^d^ (5.71)
Xinxiang	0.62 ± 0.05 ^c^ (80.5)	2.60 ± 0.07 ^b^ (4.19)	1.75 ± 0.12 ^a^ (2.82)	4.03 ± 0.05 ^c^ (6.50)
Yuzishu263	0.43 ± 0.02 ^a^ (55.8)	2.90 ± 0.16 ^b^ (6.74)	2.37 ± 0.05 ^b^ (5.51)	3.60 ± 0.12 ^b^ (8.37)
Zimei	0.50 ± 0.03 ^b^ (64.9)	1.80 ± 0.06 ^a^ (3.60)	4.12 ± 0.21 ^d^ (8.24)	2.74 ± 0.05 ^a^ (5.48)

Note: The numbers in the table represent the index of sweetness of raw sweetpotatoes and those under different cooking conditions followed by the fold of sweetness in the cooked sweetpotatoes relative to those in raw sweetpotatoes. Different lower-case letters besides the numbers in the table represent significant difference at 0.05 levels among all cultivars under the same cooking treatment.

### Effect of cooking methods on α-amylase activity in sweetpotatoes

Raw sweetpotato storage roots had the highest α**-**amylase activity while those in other treatments approached to be zero (Table 4), which indicated that cooking methods influenced amylase activity significantly. There were differences among cultivars (Table 4). Raw sweetpotatoes “*Zimei*” had the least amylase activity, and “*Jinyu*” and “*Yuzishu263*” had the highest.

**Table 4:** Effect of cooking methods on amylase activity of sweetpotato storage roots.

Variety	Raw sweetpotatoes	Baking (%)	Boiling (%)	Steaming (%)
Jinyu	12.80 ± 0.66	0.48 ± 0.03 (3.75)	0.96 ± 0.10 (7.50)	0.19 ± 0.01 (1.48)
Xinxiang	4.82 ± 0.06	0.42 ± 0.08 (8.71)	0.02 ± 0.01 (0.41)	0.00 ± 0.01 (0.00)
Yuzishu263	12.64 ± 0.20	0.00 ± 0.00 (0.00)	0.16 ± 0.01 (1.27)	0.03 ± 0.00 (0.24)
Zimei	2.23 ± 0.11	0.20 ± 0.06 (8.97)	0.07 ± 0.01 (3.14)	0.41 ± 0.09 (18.38)

Note: The numbers in the table represent α-amylase activity (mg/g of Dw/5 min) of raw sweetpotatoes and those under different cooking conditions followed by the percentage of amylase activity in the cooked sweetpotatoes relative to those in raw sweetpotatoes.

## Discussion

Our study showed that sugar composition variation was related to the color of sweetpotato flesh. In general, sweetpotato storage roots from the yellow flesh cultivars (“*Jinyu*” and “*Xinxiang*”) contained more sugars than those from the purple flesh cultivars (“*Zimei”* and “*Yuzishu263*”). Cooking increased total sugar content especially reducing sugars. This effect was different among cultivars and cooking methods. Cooking methods did not have any significant effects on non-reducing sugar content, which is in agreement with the results of Bian and Damir [24,25].

We did not cut the storage root into pieces before boiling while Lyimo et al. (2010) sliced the storage root into pieces of 3–5 cm thick then boiled (moist heat) in clean water until ready to eat (about 30–45 minutes). We considered that pieces in hot moist may lose some components into distilled water which will influence the results. For example, as to boiling, our result of reducing sugar was 75–163.6 mg/g.Dw among the four cultivars, compared to 72.02–110.4 mg/100g.Dw in Lyimo et al. (2010). As to cultivars, we selected the typical materials by their color flesh, and we supposed that different color flesh perform significantly different nutritional quality. As to starch, HClO_4_ (perchloric acid) have been employed to degrade starch, compared to method of Enzymatic hydrolysis (Thaís de Souza ROCHA et al, 2009), there was little difference. Sweetpotatoes from cultivar “*Jinyu*” had the highest soluble sugar content, the lowest starch content, the highest amylase activity and highest sweetness. Amylase activity and starch content of sweetpotatoes from other cultivars had less significant correlation with soluble sugar content. It may be related to other factors such as cooking temperature and time, as well as cultivar difference, etc. This observation was similar to that reported previously [7,25,26].

In our study, cooking decreased starch content, increased sugar content especially reducing sugars and therefore increased sweetness. This is expected since starch is degraded by enzymes and transformed into soluble sugars during cooking processes [27–29]. It was also said that α-amylase activity increased during the initial period due to the gradual increase of cooking temperature but is then significantly reduced due to the high temperature. This was demonstrated by previous study showing that decreases of reducing sugars in sweetpotatoes was due to loss of more amylase activity at 100°C by boiling and steaming than those at 80°C by baking or 40°C under sunlight [30]. However, the decrease of starch content and increase of soluble sugar content in this report is in conflict with a previous report in which boiling, roasting and sun drying did not have any significant effect on the carbohydrate, protein, fat, ash, calcium, iron and magnesium contents but decreased sugar content [8]. The different results from these two studies might be due to the differences of cooking methods, analytic methods, cultivars, etc.

Our report showed that the sweetpotatoes still had some residual amylase activities after 28 min of cooking (Table 4). The residual amount of amylase activity suggests that the sweetpotatoes were not thoroughly cooked during the initial cooking process. There may be several alternative reasons for explaining the residue activity. First, there may be extremely high temperature tolerance enzymes existing in sweetpotatoes. Second, starch degradation does not thoroughly rely on enzymes which are sensitive to temperature variation, for example, pyrolysis. Third, the final degraded products, sugars, do not completely come from starch degradation which relies on amylases, such as lipids

Our results showed that cooking decreased starch content and increased soluble sugar content, which support the well-known phenomena that cooked sweetpotato usually tastes sweeter than fresh ones. Our experiment demonstrated that different cooking methods produced different sweetness and that steaming produced the highest sweetness. Soluble sugars of cooked sweetpotatoes had higher correlation with sweetness than those of raw sweetpotatoes, suggesting that soluble sugars in cooked sweetpotatoes are more suitable than those in raw sweetpotatoes for evaluating cultivar quality during sweetpotato breeding [20].

## Conclusion

We determined the effects of cooking methods on starch, amylase and sugar components in four elite Chinese sweetpotato cultivars. Cooking reduced starch content and amylase activity and increased reducing sugar content especially maltose content as well as sweetness, but did not have significant changes in non-reducing sugar content. These effects were different among the four varieties and three cooking methods. This study provides useful information for quality analysis and cultivar appraisal of sweetpotatoes.

## Abbreviations

g: gram
mg: milligram
μg: microgram
L: liter
mL: milliliter
μL: micro liter
cm: centimeter
mm: millimeter
μm: micrometer
rpm: rotation per minute
min: minute
Dw: dry weight
Fw: fresh weight
